# Supplementary material for: Complexome profiling of the Chlamydomonas psb28 mutant reveals TEF5 as an early PSII assembly factor
Source: Plant Cell. 2025 Mar 17;37(6):koaf055. doi: 10.1093/plcell/koaf055 (PMC12164590; doi:10.1093/plcell/koaf055)
Supplement: koaf055_Supplementary_Data [file koaf055_supplementary_data.zip › psb28_interactive.zip!/plots/intensity/qualityPlots/peptides/ICL1_Cre06.g282800.t1.2.html]

### Cre06.g282800.t1.2

Trivial name: ICL1  
SeqCoverage [median]: 27.10  
Mass: 45.75 kDa  
  
 

| Sequence | MissedCleavage | Mass [Da] | wtIntensity | muIntensity | Score |
| --- | --- | --- | --- | --- | --- |
| FAAAIHAQYPGK | 0 | 1272.66 | 2212.33 | 8609.90 | 120.06 |
| GGIDAAIAR | 0 | 842.46 | 4751.46 | 8759.49 | 145.34 |
| GMSAYAQLQEAEFASEK | 0 | 1858.84 | 913.61 | 7695.21 | 156.69 |
| GSIKIEYTLAR | 1 | 1249.70 | 0.00 | 4326.72 | 88.80 |
| GSTEEEQFHH | 0 | 1199.48 | 1323.88 | 3208.90 | 126.07 |
| INNAFQR | 0 | 861.45 | 6909.79 | 13507.03 | 199.40 |
| KFAAAIHAQYPGK | 1 | 1400.76 | 0.00 | 362.36 | 35.65 |
| LAADVMDVPTLIIAR | 0 | 1596.89 | 2686.85 | 19453.12 | 142.89 |
| LSDDEIAK | 0 | 889.44 | 0.00 | 715.56 | 65.88 |
| LSDDEIAKFQK | 1 | 1292.66 | 459.91 | 0.00 | 82.17 |
| VLVPTKEFVQK | 1 | 1286.76 | 1683.17 | 9350.98 | 68.44 |
| VYTRQDVEK | 1 | 1136.58 | 0.00 | 488.46 | 64.04 |
|  |  |  | 20941.0 | 76477.7 |  |
